# Human milk oligosaccharide composition, concentrations and association with maternal factors in the multi-ethnic Asian GUSTO cohort

**DOI:** 10.3389/fnut.2026.1781871

**Published:** 2026-05-28

**Authors:** Han Zhang, Li Ting Ang, Wei Wei Pang, Charlene Wee, Chun Hong Wong, Kennedy Spann, Mei Chien Chua, Fabian Yap, Kok Hian Tan, Yap Seng Chong, Neerja Karnani, Falk Müller-Riemenschneider, Natarajan Padmapriya, Keith M. Godfrey, Peter Gluckman, Johan Eriksson, Donna Geddes, Mary E. Wlodek, Lars Bode, Shiao-Yng Chan

**Affiliations:** 1 Institute for Human Development and Potential (IHDP), Agency for Science, Technology and Research (A*STAR), Singapore, Singapore; 2 Department of Obstetrics and Gynaecology, Yong Loo Lin School of Medicine, National University of Singapore, Singapore, Singapore; 3 Global Centre for Asian Women’s Health (GloW) and Bia-Echo Asia Centre for Reproductive Longevity and Equality (ACRLE), Yong Loo Lin School of Medicine, National University of Singapore, Singapore, Singapore; 4 Department of Pediatrics, Larsson-Rosenquist Foundation Mother-Milk-Infant Center of Research Excellence (MOMI CORE), and the Human Milk Institute (HMI), University of California, San Diego, La Jolla, CA, United States; 5 Duke-NUS Medical School, Singapore, Singapore; 6 Department of Neonatology, KK Human Milk Bank, KK Women’s and Children’s Hospital, Singapore, Singapore; 7 Division of Paediatric Medicine, KK Women’s and Children’s Hospital, Singapore, Singapore; 8 Department of Obstetrics and Gynaecology, KK Women’s and Children’s Hospital, Singapore, Singapore; 9 Bioinformatics Institute (BII), Agency for Science, Technology and Research (A*STAR), Singapore, Singapore; 10 Saw Swee Hock School of Public Health, National University of Singapore, Singapore, Singapore; 11 Medical Research Council Lifecourse Epidemiology Centre, University of Southampton, Southampton, United Kingdom; 12 NIHR Southampton Biomedical Research Centre, University of Southampton and University Hospital Southampton NHS Foundation Trust, Southampton, United Kingdom; 13 Liggins Institute, University of Auckland, Auckland, New Zealand; 14 School of Molecular Sciences, UWA Centre for Human Lactation Research and Translation, The University of Western Australia, Perth, WA, Australia; 15 Department of Obstetrics, Gynaecology and Newborn Health, Faculty of Medicine, Dentistry and Health Sciences, The University of Melbourne, Melbourne, VIC, Australia

**Keywords:** Asian ethnicity, *FUT2* gene, human milk, human milk oligosaccharides (HMOs), maternal factor, secretor status

## Abstract

**Purpose:**

To investigate the human milk oligosaccharide (HMO) composition, longitudinal change and the influence of maternal factors in a multi-ethnic Asian cohort.

**Methods:**

In the prospective GUSTO mother-offspring cohort, maternal sociodemographic, genetic, and obstetric characteristics were related to the concentrations of the 19 most-abundant HMOs (quantified by HPLC, *n* = 248 mothers) at 3-weeks (*n* = 205) and 3-months (*n* = 114) postpartum (71 matched cases, 28.6%).

**Results:**

Mothers providing samples were Chinese (73.8%), Malay (14.5%) and Indian (11.7%). Across ethnicities, individual HMO concentrations and proportions of secretors, were comparable. Approximately 70% were secretors, determined by distinctly higher 2′-fucosyllactose (2’-FL, 3-weeks: 16.0% of total HMO; 3-months: 10.4%) and lacto-N-fucopentaose-I (LNFP-I), compared with non-secretors (2’-FL, 3-weeks, 0.3%; 3-months: 0.2%). 3-fucosyllactose (3-FL) comprised 9.5% and 21.7% of total HMO concentration in secretors and non-secretors, respectively, at 3-weeks, and 30.4% and 42.9%, respectively, at 3-months. Compared with term, preterm cases had higher 3′-sialyllactose (3′SL) at 3-weeks [adjusted mean difference 1.23 SDs (95%CI 0.44–2.01); *p* = 0.002]. Longitudinal changes in individual HMO concentrations from 3-weeks to 3-months were generally consistent across secretor groups. HMO-secretor-phenotype showed 97% concordance with status predicted by the *FUT2* single nucleotide polymorphism (SNP) rs1047781.

**Conclusion:**

Chinese, Malay and Indian women exhibited similar HMO compositions, with the *FUT2* rs1047781 SNP being a strong determinant of secretor status. Preterm delivery may influence specific early postpartum HMO concentrations, including a higher 3′SL concentration. The concentration of 2’-FL was lower, while 3-FL was higher, compared with published reports on non-Asians, suggesting multi-ethnic studies of infant nutrition and health should consider Asian HMO biology.

## Introduction

Human milk oligosaccharides (HMOs) are complex carbohydrates abundantly found in human milk (1). They have been shown to play a significant role in the development of the infant gut microbiome and immune system, impacting overall health (2). Therefore, identifying the factors influencing HMO composition and understanding differences between populations could offer valuable perspectives and potential strategies for optimizing infant development and wellbeing, thus, promoting a stronger foundation for future health.

There are over 200 structurally distinct HMO species, but human milk is dominated by about 20 core species, which constitute 99% of the total HMO concentration (1, 3, 4). HMOs can be categorized into three broad groupings: neutral fucosylated [e.g., 2′-fucosyllactose (2’-FL), 3-fucosyllactose (3-FL)], neutral non-fucosylated [e.g., lacto-N-tetraose (LNT), lacto-N-neotetraose (LNnT)], and acidic sialylated [e.g., 3′-sialyllactose (3′SL), 6′-sialyllactose (6′SL)] (4, 5).

The specific composition and individual concentrations of HMOs exhibit substantial inter-individual variability, influenced by genetic, environmental, and clinical factors such as pregnancy and birth complications. By HMO profiling, lactating women can largely be categorized as secretors or non-secretors, a status which is primarily determined by several single nucleotide polymorphisms (SNPs) in the maternal fucosyltransferase-2 (*FUT2*) genotype. Secretors typically have higher concentrations of fucosylated HMOs such as 2’-FL and LNFP-I. Maternal diets rich in omega-3 fatty acids, and specific prebiotics and probiotics, have been linked to the abundance of certain HMOs, while pregnancy complications including gestational diabetes mellitus (GDM), hypertensive disorders of pregnancy and preterm birth have been associated with differences in HMO profiles (6, 7). Furthermore, longitudinal studies have reported changes in HMO concentrations over the course of lactation (8, 9).

HMO composition and individual concentrations have also been reported to vary geographically, even among healthy women of similar ancestry (6). Most studies examining HMOs have focused on White, Hispanic, and Black individuals, leaving a significant gap in our understanding of HMO profiles among other ethnic groups, such as Asians, who form 60% of the global population. The few comparative studies between different ethnic groups have revealed some distinct differences in HMO profiles. For example, Asian mothers had higher LSTc and lower FDSLNH concentrations compared to Caucasian mothers living in the same geographical region, even with the same *FUT2* secretor genotype (10). Given that regional differences, encompassing variations in diet, lifestyle and socio-environmental factors, play a role in shaping HMO composition, in addition to ancestry, it is important to conduct population-specific research to understand the determinants and broader implications of population variations in HMO findings.

The Singapore demographic comprising individuals of Chinese, Malay, and Indian ethnicity presents an opportunity to compare HMO profiles among these three major Asian ethnic groups living in the same geographical location. We firstly aimed to identify potential factors associated with HMO concentrations, from sociodemographic and pregnancy/delivery characteristics. In a subset of participants providing human milk samples at both 3-weeks and 3-months post-delivery, we examined changes in individual HMO concentrations over these two timepoints. Furthermore, we sought to establish whether differences in HMO composition (i.e., relative abundance in HMOs) could be linked to specific *FUT2* SNPs that are prevalent among Asians. We aimed to provide insights that could inform the development of nutritional strategies and potential interventions to promote infant health in Asian populations.

## Methods

### Study population

The current study used data collected from the GUSTO mother-offspring cohort study (11). In brief, the GUSTO study aims to investigate the influence of early life events on health outcomes later in life. Pregnant women 18 years old and above in their first trimester were recruited from 2009 to 2010 (*n* = 1,450) with 1,181 singleton deliveries providing data for the study. Eligible participants included Singapore citizens/permanent residents with homogeneous ancestry for the three major Singaporean ethnic groups (Chinese, Malay, Indian). Women who were receiving chemotherapy, taking psychotropic drugs, or had pre-existing diabetes were excluded. In this sub-study, only mothers who provided human milk samples at either 3-weeks or 3-months postpartum were included in the analyses (*n* = 248; Supplementary Figure 1).

The GUSTO study was approved by the National Health Care Group Domain Specific Review Board (reference D/09/021) and the SingHealth Centralized Institutional Review Board (reference 2009/280/D), with written informed consent obtained at recruitment.

### Sociodemographic and clinical factors

Participant baseline characteristics, including maternal age, ethnicity, educational attainment, and pre-pregnancy alcohol consumption, were collected in the first trimester through interviewer-administered questionnaires. Maternal pre-pregnancy BMI (kg/m^2^) was derived using self-reported pre-pregnancy weight and measured height. Gestational glycemia was assessed by a 75 g oral glucose tolerance test at 26–28 weeks’ gestation and GDM status determined using the WHO 1999 criteria in clinical use at that time. Interviewer-administered structured questionnaires at 26–28 weeks’ gestation obtained maternal level of physical activity, smoking and environmental tobacco smoke exposure history, and the plasma cotinine concentration was analyzed at the same time to categorize maternal smoking status during pregnancy (12). Information on parity, hypertensive disorders of pregnancy (including pre-eclampsia, eclampsia, superimposed pre-eclampsia on chronic hypertension, pregnancy-induced hypertension), gestation at delivery, mode of delivery, whether the participant labored, child sex, and birth weight were extracted from medical records.

### Human milk collection

Milk samples were collected at 3-weeks and 3-months postpartum in participants’ homes. On the day of the home visit, mothers were instructed to: (1) refrain from feeding from 1 breast for at least 2 h before collection, (2) express milk between 6.00–10.00 a.m. into a bottle either manually or using a breast-pump until the breast was “empty,” and (3) refrigerate the bottle at 4 °C immediately. During home visits, the milk samples were warmed to 38 °C, gently mixed, and divided into 2-mL aliquots. Samples were then transported on ice back to the laboratory and stored at −80 °C until analysis.

### HMO analysis

Human milk samples were batch-analyzed at the Bode Laboratory (California, United States) in 2019. High-performance liquid chromatography (HPLC) was used to characterize HMOs, as previously described (6, 9). Briefly, human milk was spiked with raffinose (a non-HMO carbohydrate) as an internal standard to allow absolute quantification. Oligosaccharides were extracted by high-throughput solid-phase extraction over C18 and carbograph microcolumns and fluorescently labeled with 2-aminobenzamide (2AB). Labeled oligosaccharides were analyzed by HPLC on an amide-80 column (15 cm length, 2 mm inner diameter, 3 μm particle size; Tosoh Bioscience) with a 50 mmol/L ammonium formate-acetonitrile buffer system.

Separation was performed at 25 °C and monitored with a fluorescence detector at 360 nm excitation and 425 nm emission. Peak annotation was based on standard retention times and MS analysis on a Thermo LCQ Duo Ion trap MS equipped with a nano-electrospray ionization-source. Absolute concentrations were calculated based on standard response curves for each of the annotated HMOs. The total concentration of HMOs was calculated as the sum of the annotated oligosaccharides. The relative abundance of each HMO was calculated as a percentage of the total HMO concentration.

### Evaluation of *FUT2* SNPs

Maternal genotyping was performed using the Illumina OmniExpress plus Exome array. Genotype imputation was done for each ethnicity separately. Briefly, SNPs with minor allele frequency (MAF) < 5%, call rate <95% or failed Hardy–Weinberg Equilibrium at a value of *p* < 10^−6^ were excluded in each ethnic group using PLINK version 1.90. The data were aligned to GRCh37 build and further processed using a published pipeline3 before haplotype phasing using SHAPEIT2 with duoHMM method, which incorporates the family structure for better accuracy. We imputed the phased haplotypes with PBWT (Sanger Imputation Service) with the 1,000 Genomes Phase 3 as reference (13). Among 6,978,879 SNPs that passed stringent quality control (MAF > 5% and imputation INFO>0.50) in at least one ethnicity, three SNPs in the *FUT2* gene were identified (rs1047781, rs1800027, rs16982241) and investigated for concordance with the participants’ actual phenotype of secretor status as defined by HMO concentrations.

### Statistical analyses

All analyses were conducted in Python (version 3.9.16), with statistical computations performed using the Pandas, NumPy, and Statsmodels libraries (14–16). The analysis was managed within a Conda environment,^1^ and packages were sourced from the conda-forge community repository (Conda-forge, 2021). There was no imputation for missing data.

To define secretor status, we used the concentrations of 2′-fucosyllactose (2’-FL) and lacto-N-fucopentaose I (LNFP-I) assessed as their relative abundance among all measured HMOs, which are indicative of functional FUT2 enzyme activity. We firstly identified secretors using the well-accepted criterion of a 2’-FL relative abundance above 10%, while non-secretors were identified by a near-zero abundance (<0.5%) (9, 17, 18). For intermediate cases lying between these thresholds, we performed a Receiver Operating Characteristic (ROC) analysis using the clearly defined secretors and non-secretors as reference cases to determine the optimal LNFP-I relative abundance cutoff. By maximizing Youden’s Index, we identified 4% as the optimal LNFP-I threshold to discriminate between secretors (LNFP-I > 4%) and non-secretors (LNFP-I < 4%).

Comparisons of categorical variables between secretors and non-secretors were assessed using chi-squared tests. We used linear regression models to assess the association between each candidate maternal/pregnancy/delivery factor (independent variables) and the concentration of each HMO at 3-weeks and 3-months (dependent variables). For regression, all HMO concentrations were log-transformed and standardized (converted to z-scores) to correct for non-normal distributions and allow comparisons between HMOs with vastly different absolute concentrations. All candidate factors were used as categorical variables (defined in Table 1). Where associations were found in unadjusted univariate analyses, models were further adjusted for maternal age, education, pre-pregnancy BMI, parity, and infant sex. When investigating links with preterm delivery, additional adjustment was made for hypertensive disorders as a potential confounder. Additionally, the association of 3-week HMO concentrations (as independent predictor variable) and ongoing breastfeeding at 3-months (as the dependent outcome binary variable) was determined using logistic regression. False discovery rate (FDR) correction was applied using the Benjamini-Hochberg (BH) method to account for multiple comparisons. Regression results are reported as adjusted *β* coefficients with 95% confidence intervals (CI) and corresponding *p*-values, and are visualized using heatmaps to illustrate the magnitude and direction of the associations.

**Table 1:** Participant characteristics.

Variables, n (%)	All participants^a^ (*n* = 248)	Secretors^b^ (*n* = 179)	Non-secretors^b^ (*n* = 69)
Ethnicity			
Chinese	183 (73.8%)	132 (73.7%)	51 (73.9%)
Malay	36 (14.5%)	27 (15.1%)	9 (13.0%)
Indian	29 (11.7%)	20 (11.2%)	9 (13.0%)
Pre-pregnancy BMI (in kg/m^2^), Asian categories			
Underweight (<18.5)	27 (11.6%)	19 (11.4%)	8 (12.1%)
Normal (18.5– < 23)	129 (55.6%)	93 (56.0%)	36 (54.5%)
Overweight (23– < 27.5)	52 (22.4%)	37 (22.3%)	15 (22.7%)
Obese (≥27.5)	24 (10.3%)	17 (10.2%)	7 (10.6%)
Maternal level of physical activity before pregnancy			
<600 MET-week	47 (19.3%)	34 (19.4%)	13 (19.1%)
600–3,000 MET-week	131 (53.9%)	94 (53.7%)	37 (54.4%)
3,000 + MET-week	65 (26.7%)	47 (26.9%)	18 (26.5%)
Maternal level of physical activity during pregnancy			
<600 MET-week	87 (35.7%)	60 (34.1%)	27 (39.7%)
600–3,000 MET-week	111 (45.5%)	83 (47.2%)	28 (41.2%)
3,000 + MET-week	46 (18.9%)	33 (18.8%)	13 (19.1%)
Maternal smoking during pregnancy			
Non-smoker	149 (65.4%)	107 (64.5%)	42 (67.7%)
Plasma cotinine undetected but current smoker and/or ETS exposure	58 (25.4%)	44 (26.5%)	14 (22.6%)
Plasma cotinine detectable, ≥0.17 μg/L	21 (9.2%)	15 (9.0%)	6 (9.7%)
Pre-pregnancy alcohol consumption			
No	148 (60.2%)	110 (61.8%)	38 (55.9%)
Yes	98 (39.8%)	68 (38.2%)	30 (44.1%)
Parity			
Nulliparous	119 (48.0%)	86 (48.0%)	33 (47.8%)
Parous	129 (52.0%)	93 (52.0%)	36 (52.2%)
Gestational Diabetes (WHO-1999 definition^&^)			
No	198 (83.5%)	145 (84.3%)	53 (81.5%)
Yes	39 (16.5%)	27 (15.7%)	12 (18.5%)
Fasting plasma glucose at 26–28 weeks of pregnancy			
Normal (<5.1 mmol/L)	229 (96.6%)	165 (95.9%)	64 (98.5%)
High (≥5.1 mmol/L)	8 (3.4%)	7 (4.1%)	1 (1.5%)
2 h plasma glucose (after 75 g glucose load) at 26–28 weeks of pregnancy			
Normal (<8.5 mmol/L)	214 (90.3%)	157 (91.3%)	57 (87.7%)
High (≥8.5 mmol/L)	23 (9.7%)	15 (8.7%)	8 (12.3%)
Hypertensive disorders of pregnancy^#^			
No	233 (93.3%)	171 (96.1%)	62 (89.9%)
Yes	14 (5.7%)	7 (3.9%)	7 (10.1%)
Combined labor and mode of delivery			
Non-labor cesarean section	27 (10.9%)	17 (9.5%)	10 (14.5%)
Intrapartum cesarean section	34 (13.7%)	26 (14.5%)	8 (11.6%)
Vaginal delivery	187 (75.4%)	136 (76.0%)	51 (73.9%)
Gestational age at birth			
Preterm (<37 weeks)	9 (3.6%)	6 (3.4%)	3 (4.3%)
Early term (37^+0^ weeks to 38^+6^ weeks)	100 (40.3%)	79 (44.1%)	21 (30.4%)
Term (39^+0^ weeks to 41^+6^ weeks)	139 (56.0%)	94 (52.5%)	45 (65.2%)
Sex of child			
Male	128 (51.6%)	102 (57.0%)	26 (37.7%)
Female	120 (48.4%)	77 (43.0%)	43 (62.3%)
Birthweight percentile (standardized for sex and gestation by local reference)			
Small-for-Gestational-Age (SGA, <10^th^ centile)	21 (8.5%)	13 (7.3%)	8 (11.6%)
Appropriately-Grown-for-Gestational-Age (AGA, 10-90^th^ centile)	180 (72.6%)	131 (73.2%)	49 (71.0%)
Large-for-Gestational-Age (LGA, >90^th^ centile)	47 (18.9%)	35 (19.5%)	12 (17.4%)
Full breastfeeding^1^ at 1 month			
No	148 (60.9%)	101 (57.7%)	47 (69.1%)
Yes	95 (39.1%)	74 (42.3%)	21 (30.9%)
Full breastfeeding at 3 months^^^			
No	161 (66.0%)	109 (61.9%)	52 (76.5%)
Yes	83 (34.0%)	67 (38.1%)	16 (23.5%)
Any breastfeeding^2^ at 3 months			
No	42 (17.6%)	26 (14.9%)	16 (24.6%)
Yes	197 (82.4%)	148 (85.1%)	49 (75.4%)

a Percentages for the variables total 100% column-wise.

b Percentages by secretor status total 100% column-wise.

# includes pre-eclampsia, eclampsia, superimposed pre-eclampsia on chronic hypertension, pregnancy-induced hypertension.

1 Full breastfeeding includes those who were exclusively or predominantly breastfeeding, with no intake of solids; any method of breastmilk feeding is included (direct from the breast or expressed milk given through a bottle).

2 Any breastfeeding includes those who were breastfeeding, regardless of the extent of breastfeeding or the method of breastmilk feeding.

& WHO-1999 definition of gestational diabetes with a 75 g two-time point oral glucose tolerance test: fasting plasma glucose ≥7.0 mmol/L; 2-h plasma glucose ≥7.8 mmol/L.

^Restricted to cases with milk samples collected at 3-months. MET-week; Metabolic Equivalent Task per week. Due to missing data, totals may not sum to the sample size shown at the top of each column.

For the subset of participants who provided matched samples at both 3-weeks and 3-months postpartum, Spearman correlation of the absolute HMO concentrations between the two timepoints were conducted and visualized as scatter plots for each HMO. Moreover, changes in HMO concentrations over the two timepoints, as well as differences in HMO concentrations following stratification by secretor status were evaluated with the Wilcoxon signed-rank test and presented as boxplots.

## Results

248 lactating mothers provided milk samples for HMO analysis at either 3-weeks (*n* = 205) or 3-months (*n* = 114) postpartum, with 71 mothers (28.6%) providing samples at both timepoints. The characteristics of the 248 mother–child dyads are summarized in Table 1. Participants were mainly of Chinese ethnicity (73.8%), with others of Malay (14.5%) or Indian (11.7%) ethnicity. Approximately half of the participants were first-time mothers. Most mothers had uncomplicated pregnancies, with a minority having hypertensive disorders of pregnancy (6%), gestational diabetes (WHO 1999 criteria; 16%), and elevated fasting (≥5.1 mmol/L; 3%) and 2-h (≥8.5 mmol/L; 10%) glycemia in a mid-gestation oral glucose tolerance test. Approximately half the infants were boys (51.6%) and most were born at term (≥37 weeks’ gestation, 96.4%) with the majority being appropriately-grown-for-gestational age at birth (69.0%). At 3-months, 82.4% of the infants were still receiving some breastmilk.

### Secretor status

Out of the 248 mothers, 110 (44.4%) were clearly secretors (2’-FL concentrations above 10% of the total HMO concentration) and 62 (25%) clearly non-secretors (2’-FL concentrations below 0.5% of the total HMO concentration). There were 76 samples (30.6%) with an intermediate 2’-FL phenotype (concentration range 0.5 to 9.8%). Among these intermediate cases, the assignment of secretor status then considered another HMO that is also dependent on FUT2 activity; where LNFP-I concentrations constituted >4% of the total HMO concentration, mothers were classified as secretors, while the remainder were classified as non-secretors. Finally, 179 (72%) were categorized as secretors and 69 (28%) as non-secretors based on both the relative abundance of 2’-FL and LNFP-I. The characteristics of participants providing breastmilk samples at 3-weeks and 3-months stratified by secretor status are described in Supplementary Table 1.

**Figure 1:**
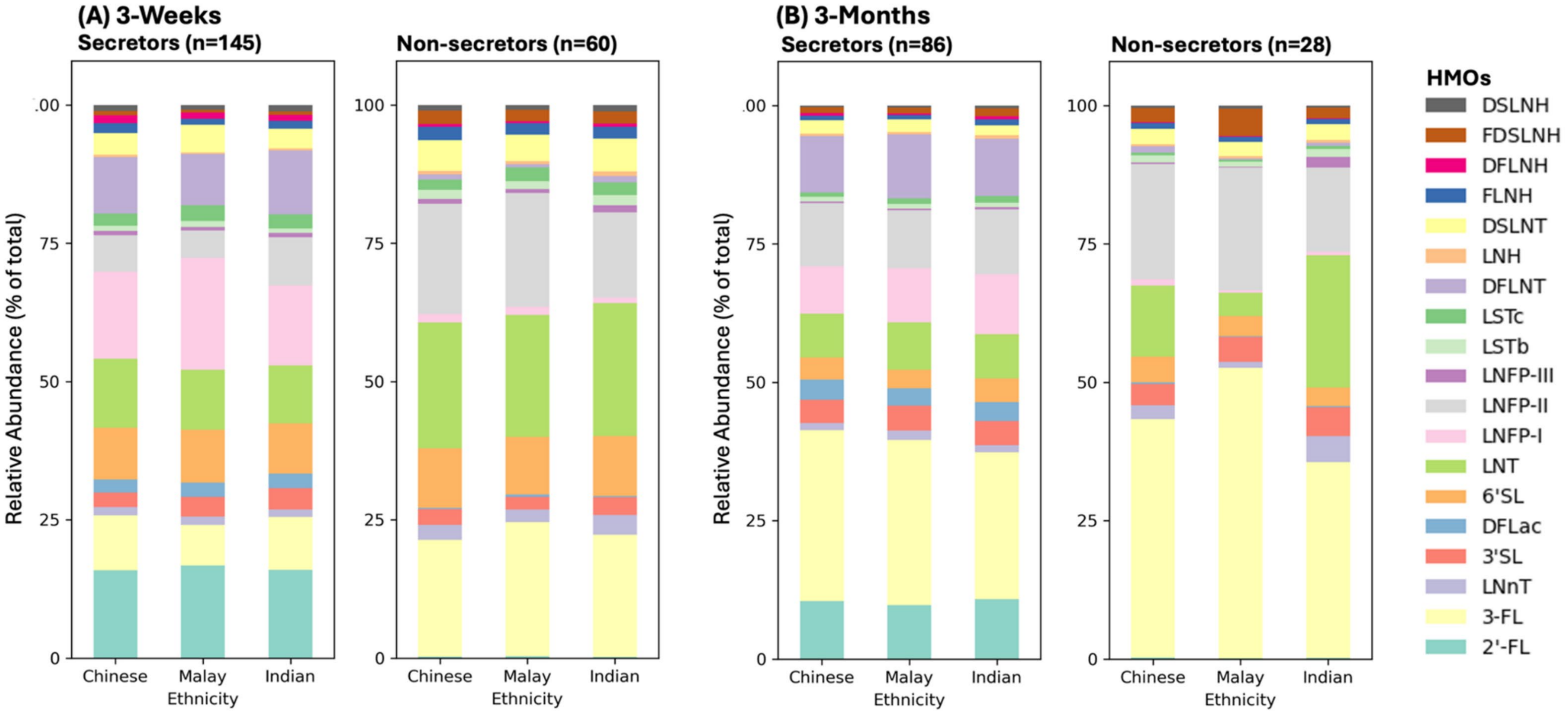
Human milk composition represented by the relative abundance of individual HMOs at 3-weeks **(A)** and 3-months **(B)** across three ethnicities stratified by secretor status. Abbreviations: 2’-FL, 2′-fucosyllactose; 3-FL, 3-fucosyllactose; LNnT, lacto-N-neotetraose; 3’SL, 3′-sialyllactose; DFLac, difucosyllactose; 6’SL, 6′-sialyllactose; LNT, lacto-N-tetraose; LNFP, lacto-N-fucopentaose; LSTb, sialyllacto-N-tetraose b; LSTc, sialyllacto-N-tetraose c; DFLNT, difucosyllacto-N-tetraose; LNH, lacto-N-hexaose; DSLNT, disialyllacto-N-tetraose; FLNH, fucosyllacto-N-hexaose; DFLNH, difucosyllacto-N-hexaose; FDSLNH, fucosyldisialyllacto-N-hexaose; DSLNH, disialyllacto-N-hexaose.

Secretors displayed substantially higher concentrations and relative abundance of 2’-FL, LNFP-I, DFLac, DFLNT and DFLNH as compared to non-secretor mothers (Figure 1). Absolute concentrations are presented for secretor and non-secretor groups in Supplementary Table 2. There were distinctly higher 2’-FL (mean ± SEM 2391 ± 137 nmol/mL, 16.0% of total HMO at 3-weeks; 1,677 ± 114 nmol/mL, 10.4% at 3-months) and LNFP-I (2,441 ± 99 nmol/mL, 16.4% at 3-weeks; 1,423 ± 127 nmol/mL, 8.9% at 3-months) concentrations compared with non-secretors (2’-FL: 29 ± 5 nmol/mL, 0.3% at 3-weeks; 28 ± 4 nmol/mL, 0.2% at 3-months; LNFP-I: 151 ± 8 nmol/mL, 1.4% at 3-weeks; 120 ± 15 nmol/L, 1.0% at 3-months). Another abundant HMO in our population, 3-fucosyllactose (3-FL), comprised 9.5% and 21.7% of the total HMO concentration in secretors and non-secretors, respectively, at 3-weeks, and 30.4% and 42.9%, respectively, at 3-months.

**Figure 2:**
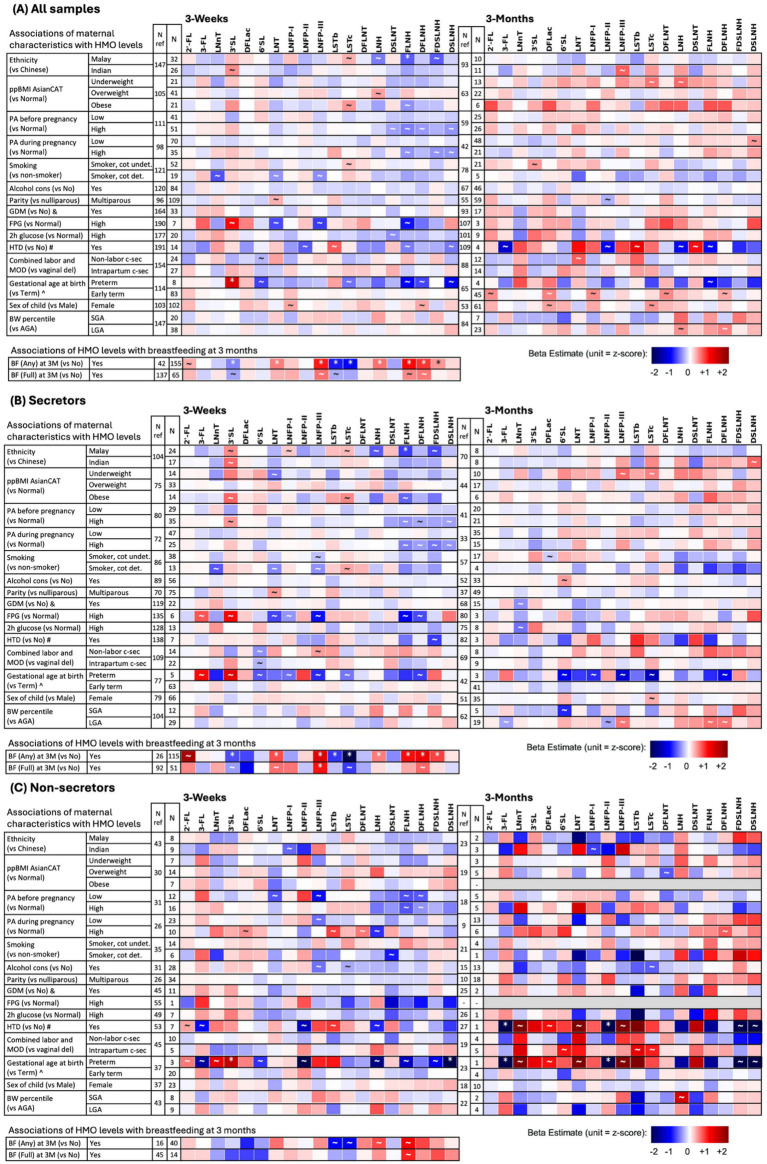
Maternal factors in relation to HMO concentrations at 3-weeks and 3-months in a combined secretor/non-secretor group (all; **A**) and separately in secretor **(B)** and non-secretor **(C)** groups (defined by the relative abundance of 2’-FL and LNFP-I). Log transformed and standardized across both time points. Beta estimates from unadjusted univariate linear regression. ~Uncorrected *p* < 0.05, *FDR-corrected (BH) *p* < 0.05. ^Preterm birth is defined as delivery before 37 weeks + 0 days gestation; Early term is defined as delivery between 37 weeks + 0 days and 38 weeks + 6 days gestation; Term is defined as delivery at or after 39 weeks + 0 days gestation. ^#^Hypertensive disorders of pregnancy includes pre-eclampsia, eclampsia, superimposed pre-eclampsia on chronic hypertension, pregnancy-induced hypertension. ^&^Gestational diabetes mellitus is defined by the WHO 1999 criteria. High fasting glucose is defined as ≥5.1 mmol/L at 26–28 weeks of pregnancy. High 2 h glucose is defined as ≥8.5 mmol/L following a 75 g glucose load in an oral glucose tolerance test at 26–28 weeks of pregnancy. Abbreviations: Alcohol cons, alcohol consumption during pregnancy; BF (Any), Any breastfeeding; BW percentile, birth weight standardized for sex and gestational age by local references; C-section, cesarean section; GDM, Gestational Diabetes Mellitus; FPG, fasting plasma glucose at mid-gestation; HTD, hypertensive disorders of pregnancy including pre-eclampsia, eclampsia and pregnancy-induced hypertension; MOD, Mode of delivery; Non-Smoker, plasma cotinine undetectable in, self-reported non-smoker with no exposure to environmental tobacco smoke; Smoker, plasma cotinine detectable (cot det.) and/or self-reported smoker or exposure to environmental tobacco smoke; SGA, small-for-gestational-age; AGA, appropriately-grown-for-gestational-age; LGA, large-for-gestational-age, LGA; ppBMI, pre-pregnancy BMI; PA, level of physical activity; U/w labor, underwent labor.

Between the three Asian ethnic groups, there were no differences in the proportion classified as secretors (Chinese: 72.1%, Malay: 75.0%, Indian: 69.0%; Chi-squared *p* = 0.864). In addition, among the 8 most abundant HMOs, there were no ethnic differences in concentrations when stratified by secretor status (Figures 2B,C). Participant characteristics were largely similar between the two secretor status groups (Table 1), except a slightly higher proportion of non-secretors had a hypertensive disorder of pregnancy (*p* = 0.058), delivered a female infant (*p* = 0.006), and ceased full breastfeeding at 3-months (*p* = 0.032), but none of these associations remained following FDR-correction, suggesting findings may be spurious.

### Factors contributing to HMO variance among Asians

#### Ethnicity

Combining secretors and non-secretors in unadjusted analyses (Figure 2A), compared to Chinese, Malays had a lower concentration of FLNH at 3-weeks postpartum, which was no longer observed at 3-months. This association was observed only among secretors (see Figure 2B), where with the adjustment for key covariates, mean FLNH at 3-weeks was −0.74 SDs (95%CI −1.20, −0.28; *p* = 0.002) lower in Malays compared with Chinese.

#### Preterm delivery

In combined secretor/non-secretor unadjusted analyses (Figure 2A), delivery preterm was associated with a higher 3’SL concentration at 3-weeks postpartum compared with delivery at term. With adjustment for covariates and hypertensive disorders of pregnancy, the 3’SL concentration remained higher in samples from mothers delivering preterm compared with those from mothers who delivered early term and at term combined [8 preterm vs. 197 term; adjusted mean difference 1.23 SDs (95%CI 0.44, 2.01); p = 0.002], with this association being more prominent among non-secretors [1.80 SDs (0.78, 2.81); *p* = 0.001] (Figure 2C) than among secretors, where a similar trend was found (Figure 2B). When stratified by secretor status, other HMO associations with preterm birth were observed among non-secretors (Figure 2C): lower DSLNH concentration [−3.08 SDs (−4.73, −1.42); *p* < 0.001] at 3-weeks (3 preterm vs. 57 term). However, the apparently lower 3-FL and LNFP-II concentrations at 3-months in the sole preterm birth case providing data (1 preterm vs. 27 term) were found in adjusted analysis to be confounded by hypertensive disorder of pregnancy. With small sample sizes cautious interpretation is required.

#### Hypertensive disorders of pregnancy

Hypertensive disorders of pregnancy were not associated with persistent and consistent alterations in HMOs across timepoints in combined secretor/non-secretor unadjusted analyses (Figure 2A). The few differences observed among non-secretors (Figure 2C) at 3-months of lower 3-FL and LNFP-II concentrations compared with pregnancies without hypertensive disorders were driven by data provided by the sole case of hypertensive disorder in pregnancy that also delivered preterm and is therefore unreliable.

#### Other factors

There were no clear associations between gestational diabetes, mid-gestation fasting glucose and post-load glucose concentrations, pre-pregnancy BMI, lifestyle factors (physical activity, smoking/environmental smoke exposure), delivery events (undergoing labor, mode of delivery) and infant sex with any HMO at 3-weeks and 3-months in univariate analyses combining secretor/non-secretor groups as well as in stratified analyses by secretor status (Figures 2A–C).

### Associations of HMO concentrations at 3-weeks and breastfeeding duration

Several HMOs at 3-weeks were observed to predict any ongoing breastfeeding at 3-months (Figure 2A), specifically higher LNT, LNFP-III, LNH, FLNH, DFLNH, FDSLNH, and lower 3’SL, LSTb, LSTc. This was especially observed among secretors (Figure 2B). When considering the outcome of specifically full breastfeeding, defined as exclusive or predominant breastfeeding, at 3-months a significant association was observed only among secretors, where a higher LNFP-III concentration at 3-weeks was associated with full breastfeeding at 3-months (Figure 2B).

### Correlations between different HMOs

**Figure 3:**
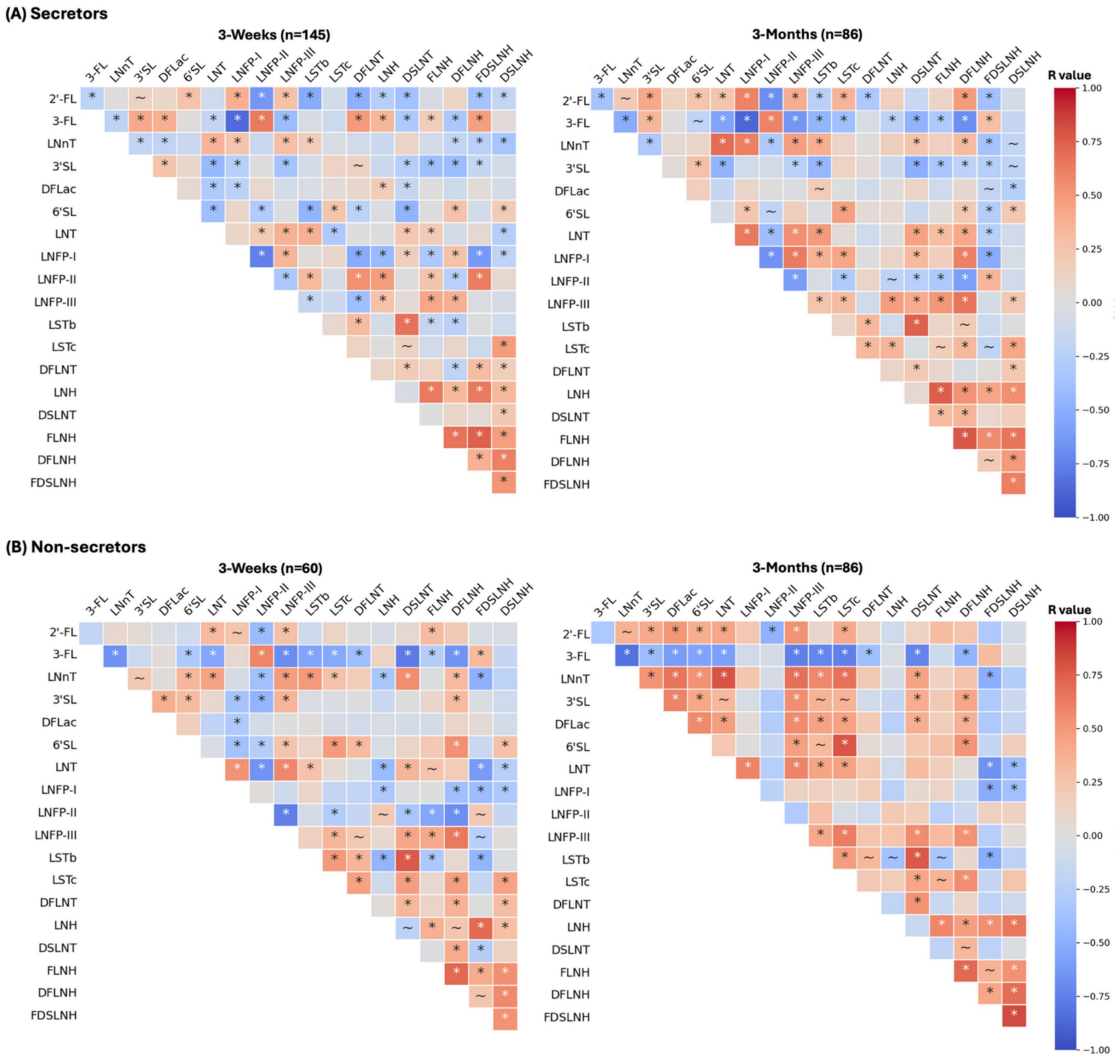
Correlations between different HMO concentrations at 3 weeks and at 3 months stratified by secretor status (defined by the relative abundance of 2’-FL and LNFP). ~0.05 < *p* < 0.1, *p < 0.05.

The general pattern of correlations between different HMOs at 3-weeks remained consistent at 3-months but with some changes in the magnitude of the correlation (Figure 3). The concentrations of the FUT2-dependent HMOs, 2’-FL and LNFP-I, were correlated with each other at both timepoints while other HMOs commonly correlated with 2’-FL in literature such as DFLac, LNH, and DSLNH were either not correlated or inversely correlated with 2’-FL and LNFP-I instead in our cohort. The concentrations of the FUT3-dependent HMOs, 3-FL and LNFP-II, were correlated with each other as expected.

### Changes in HMO concentrations from 3-weeks to 3-months

**Figure 4:**
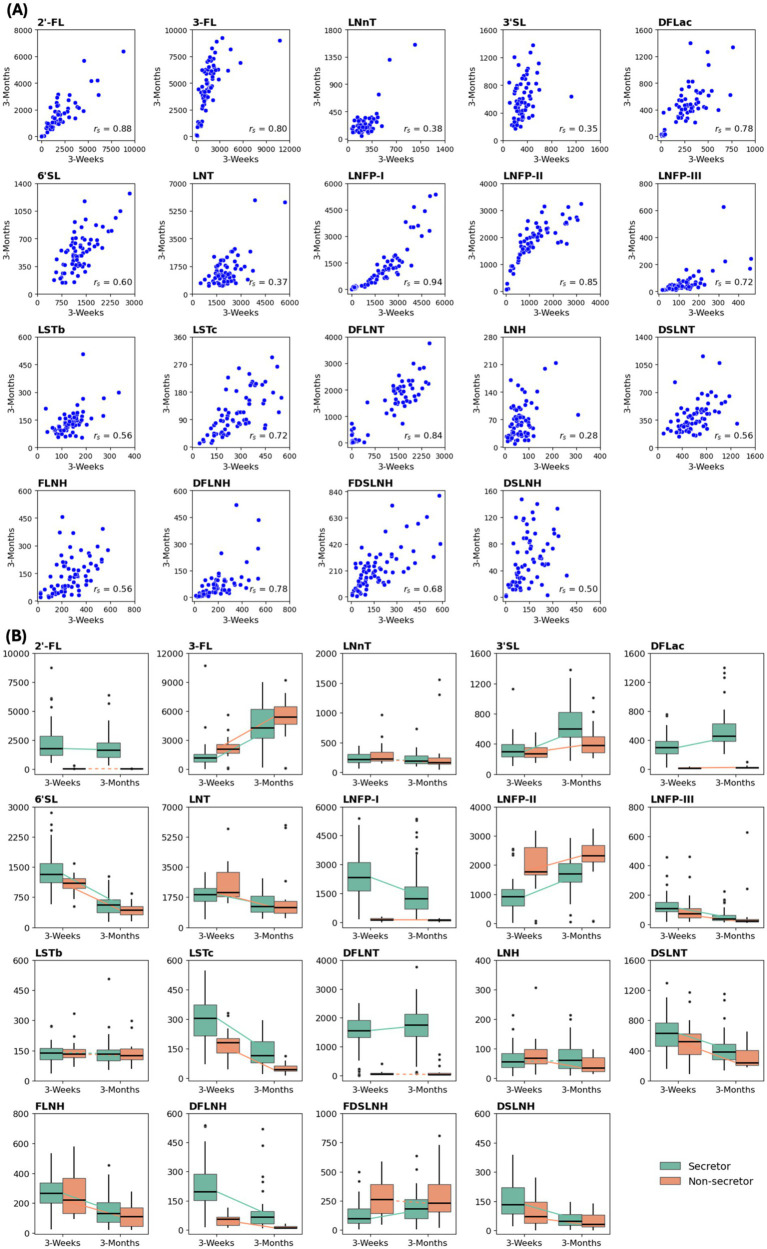
Individual HMO concentrations correlated between 3 weeks and 3 months **(A)**, and when stratified by secretor status **(B)** (defined by the relative abundance of 2’-FL and LNFP) among those providing samples at both timepoints (*n* = 71). Spearman correlation was used to assess the correlation between individual HMO concentrations at 3 weeks and 3 months; all correlation coefficients (*r*_ss_) in **(A)** were statistically significant (*p* < 0.05). The Wilcoxon signed-rank test was used to evaluate changes in HMO concentrations between the two time points. In **(B)**, solid lines indicate statistically significant changes (*p* < 0.05), while dashed lines indicate non-significant changes.

There were generally strong correlations observed between the concentration of each individual HMO measured at 3-weeks with the corresponding concentration at 3-months (see Figure 4A; all *p* < 0.05). The strongest correlations (*r_s_* > 0.75) across these two timepoints were observed for the following HMOs: 2’-FL, 3-FL, DFLac, LNFP-I, LNFP-II, DFLNT, and DFLNH.

However, the absolute HMO concentrations often changed substantially from 3-weeks to 3-months postpartum (Figure 4B). Some HMO concentrations increased (e.g., 3-FL, 3’SL, LFNP-II; *p* < 0.05) while others decreased (e.g., 6’SL, LNFP-III, LSTc, DSLNT, FLNH; *p* < 0.05). The direction of change were mostly similar among secretors and non-secretors, except for DFLac (increased in secretors but remained low in non-secretors), LNFP-I (decreased in secretors but remained low in non-secretors) and FDSLNH (slight increase in secretors but remained high in non-secretors).

### Links with the *FUT2* genotype

Three SNPs in the *FUT2* gene were available for investigation (rs1047781, rs1800027, rs16982241). For the *FUT2* rs1047781 SNP, only 3% of cases showed clear discordance with the binary secretor phenotype as determined by the relative abundance of 2’-FL and LNFP-I, with the remaining 97% showing concordance with either the predicted phenotype or an intermediate phenotype (Table 2). All of the 7 discordant cases (3%) were those with a supposedly secretor genotype but who showed a non-secretor HMO profile. Of those with the A_A SNP (37% of cohort; secretor genotype), 56 cases displayed clear secretor phenotype, 25 cases an intermediate phenotype (2’-FL concentration 5.0–9.8%; based on LNFP-I 24 finally classed as secretors, 1 as non-secretor) and only 6 cases displayed a clear non-secretor phenotype according to the HMO profile. Among those with the T_T SNP (21% of cohort; non-secretor genotype), 43 showed the expected non-secretor HMO profile, while 6 had an intermediate phenotype (all finally classified as non-secretors) and none had a secretor phenotype. Among those with the T_A SNP (41% of cohort; intermediate genotype), 50 showed a clear secretor HMO phenotype, 40 had an intermediate phenotype (all finally classed as secretors), while 7 cases had a clear non-secretor phenotype. The two other available *FUT2* SNPs, rs1800027 and rs16982241 (Table 2), were non-discriminatory for secretor phenotype.

**Table 2:** Alignment of secretor status determined by the relative abundance of 2’-FL in human milk with the three available SNPs in the *FUT2* gene.

Secretor status determined by 2’-FL abundance^	SNPs in the *FUT2* gene		
	*rs1047781*		
	A_A	T_A	T_T
Secretor	56 (24%)	50 (21%)	0 (0%)
Intermediate	25 (11%)	40 (17%)	6 (3%)
Non-secretor	6 (3%)	7 (3%)	43 (18%)
	*rs1800027*		
	A_A	G_A	/
Secretor	101 (43.2%)	5 (2%)	/
Intermediate	70 (30%)	1 (0.4%)	/
Non-secretor	57 (24.4%)	0 (0%)	/
	*rs16982241*		
	A_A	A_G	G_G
Secretor	3 (1.3%)	31 (13.3%)	72 (31%)
Intermediate	1 (0.4%)	7 (3%)	63 (27%)
Non-secretor	0 (0%)	0 (0%)	56 (24%)

**^**Secretor: 2’-FL >10%; Intermediate: 2’-FL between 0.05 and 10%; Non-secretor: 2’-FL <0.5%. Abbreviations: SNP, single nucleotide polymorphism; A, adenine; G, guanine; T, thymine.

## Discussion

This study of HMOs is the first to include the three major Asian ethnic groups of Chinese, Malay and Indian, providing insights on the potential influence of ancestry and culture while minimizing the confounding of geography since all participants were living within the same small, urbanized environment of Singapore. The proportion of secretors (70%) and non-secretors (30%) was similar across the three Asian ethnicities, with only the concentration of one HMO, FLNH, showing an ethnic difference of being lower in Malays compared with Chinese. Associations of preterm delivery of the recently ended pregnancy with a higher 3’SL concentration at 3-weeks post-delivery, more marked among non-secretors, suggest that early untimely delivery could influence individual HMO concentrations, possibly due to interruption of antenatal breast development. There were generally strong correlations between individual HMO concentrations at 3-weeks with corresponding concentrations at 3-months, in keeping with our present understanding that HMO composition is primarily driven by stable factors such as genotype. Of interest, the concentrations of a set of HMOs at 3-weeks could predict ongoing breastfeeding at 3-months and may represent biomarkers of improved milk production. Consistent with previous reports, the *FUT2* genotype at the rs1047781 SNP is a strong determinant of secretor status in Asians (19, 20). These findings offer important insights into the main clinical and genetic factors influencing HMO profiles among Asians, which may have potential implications for breastfeeding practices and customized strategies to promote infant health outcomes.

Typically in published studies, secretor status can be distinguished with a high degree of clarity: non-secretors show 2’-FL concentrations below 0.5% of the total HMO concentration, whereas secretors display concentrations above 10%. However, a third of our cohort showed intermediate 2’-FL levels, complicating this binary classification. Similar findings have been observed in other Asian cohorts such as lactating mothers in Indonesia and mainland China (19, 21), consistent with the suggestion that the presence of specific *FUT2* SNPs among Asians, while not causing complete loss of function, could reduce enzymatic activity (instead of their near absence) resulting in lower concentrations of 2’-FL and LNFP-I (22). The *FUT2* gene encodes the alpha-1,2-L-fucosyltransferase enzyme involved in the synthesis of 2’-FL and related HMOs. It has been postulated that partial enzyme activity may be linked to region-specific SNPs that do not introduce premature stop codons but rather decrease overall enzyme function, leading to atypical HMO profiles (6, 10). This may partly explain the lack of correlation between 2’-FL and another *FUT2*-dependent HMO, DSLNH, in our cohort since DSLNH may require a higher level of enzyme activity for its synthesis compared with the more abundant 2’-FL (20).

The high concordance between HMO-defined secretor status and *FUT2* rs1047781 SNP variation aligns with previous findings (19). A study confirmed the ability of this *FUT2* SNP to bring about substantial changes in *FUT2* enzyme activity to determine HMO secretor phenotype (23). However, in the non-concordant cases (3%) it is likely that other *FUT2* SNPs or possibly gene expression or protein function modulators are at play to reduce the influence of rs1047781 on enzyme activity. However, the *FUT2* SNPs rs1800027 and rs16982241 appear non-discriminatory of secretor status, with no previous study reporting on these SNPs.

While our findings suggest minimal ethnic variation in HMO profiles within this multi-ethnic Asian population in Singapore, comparing our results to other studies on non-Asian cohorts revealed marked inter-population differences. For instance, compared to North American, European and African cohorts (6), our cohort exhibited lower 2′-FL and higher 3-FL concentrations, aligning more closely with Asian findings from mainland-Chinese and Indonesian-Malay populations (19, 24, 25). Moreover, geographical variation in the proportion of secretors and non-secretors have been reported, with the Peruvian and Mexican populations reporting as high as 98–99% being secretors while most Caucasian studies found about 70–80% were secretors (6, 26). Our study’s similarities in the proportions of secretors among Chinese, Malay, and Indian ethnicities may be due to their relatively close common ancestry. Further, genetic variations in the *FUT2* and *FUT3* genes across populations have not always translated into significant variations in HMO profiles across ethnic groups (27) and it is likely that a combination of specific key SNPs may underlie HMO variation. We cannot, however, exclude the possibility that with our smaller sample size of Malay and Indian mothers we were underpowered to determine ethnic differences within our cohort. In our study, the only notable ethnic difference was a lower concentration of FLNH among Malay mothers compared to Chinese mothers, particularly at 3-weeks post-delivery among secretors. This difference was not consistently observed at 3-months post-delivery, suggesting it may be a spurious association. Nonetheless, we cannot entirely rule out the influence of culturally-specific postpartum practices, such as the confinement diet that is practized only during the first month postpartum, varying between ethnic groups in Singapore, which may impact human milk composition through nutritional or metabolic pathways (28).

A few studies have explored the relationship between HMOs and complications in the recently ended pregnancy such as preterm birth, hypertensive disorders of pregnancy and gestational diabetes mellitus (GDM). Similar to our findings, one study had reported increased 3’SL concentrations at 2–8 weeks after a preterm delivery (29). Studies also reported higher concentration of LSTs, LSTb, LSTc, and DSLNT (29, 30) but these were not observed in our cohort but rather a lowered concentration of DSLNH was found. Changes in HMO composition following preterm birth has been postulated to assist in preterm neonatal adaptation to promote healthy development (7), but we hypothesize that it is more likely a consequence of less mature antenatal breast development, with 3’SL being the dominant one affected and the common one between different populations.

Our possibly unreliable finding of lower 3-FL and LNFP-II concentrations with hypertensive disorders, based on a single case among non-secretors at 3-months, contrasts those from a study in a mainland Chinese population which reported, instead, trends of higher levels of fucosylated HMOs in the initial breast colostrum (31). Other studies reported no associations between hypertensive disorders and HMO profiles but they were mainly conducted in populations with a high proportion of secretors among whom these HMO alterations may be less marked (32). Larger studies including more non-secretors are required to confirm if hypertensive disorders of pregnancy really influence HMO composition. Since hypertensive disorders are among the major indications for medically-induced preterm delivery, these clinical factors may confound changes in HMO profiles and the lack of consideration of both factors simultaneously could underlie inconsistent results between studies. The mechanisms of how preterm delivery and hypertensive disorders could influence HMO composition remain unknown and is an area worthy of further investigation in attempts to optimize infant outcomes in such scenarios. We have previously linked hypertensive disorders with potentially compromised milk production (33), and this may be one mechanism by which HMO composition could be altered, however, in our present study neither 3-FL nor LNFP-II were among the HMOs at 3-weeks that were predictive of ongoing breastfeeding at 3-months.

Reports on links between GDM and HMO composition are inconsistent with some studies reporting that certain HMOs, such as LNnT, were significantly higher in mothers who had GDM (34), while other studies (35) reported no associations between maternal glycemia and HMO concentrations like our study. The differential exposure to and the relative strength of impact of the many confounding factors that could affect both GDM risk and HMOs in different populations, may explain these inconsistent observations.

The strong correlation between HMOs at 3-weeks and 3-months after delivery and the similar patterns of change in secretors and non-secretors align with literature on HMO stability, believed to be driven largely by maternal genotype. A study (29) observed that individual HMO relative abundance are generally stable over the early postpartum period, supporting our results that general HMO composition maintain consistency over the first 3 months postpartum. Of note, only mothers who continued breastfeeding at 3-months were able to provide our study with samples for longitudinal comparisons. Despite such consistency, we found that there were HMO variations that could predict breastfeeding continuation. Our finding of the association between higher levels of LNT, LNFP-III, LNH, FLNH, DFLNH, and FDSLNH, and lower levels of 3’SL, LSTb and LSTc at 3-weeks with ongoing breastfeeding at 3-months suggests that these oligosaccharides might reflect aspects of milk production and breastfeeding practices, and therefore represent potential biomarkers of sufficient breastmilk production leading to a longer breastfeeding duration. In particular, among secretors, the LNFP-III concentration at 3-weeks was predictive of full-breastfeeding at 3 months. However, we cannot exclude the possibility that these associations were confounded by a greater commitment to breastfeeding resulting in more frequent breastfeeding at 3-weeks, which in turn, leads to both altered HMO concentrations and independently to longer breastfeeding duration.

The main novelty of this two timepoint study of HMOs within the GUSTO cohort is the contemporaneous collection and simultaneous assessment of breastmilk among the three major Asian ethnic groups, adding insights to this less studied segment of the global population. The study population lived within a small island in mixed-race neighborhoods, therefore minimizing confounding by geographical location. Our study, however, has several limitations. The majority of the participants were Chinese with modest numbers of Malay (*n* = 36; 14.5%) and Indian (*n* = 29; 11.7%) participants, thus larger, more ethnically-balanced studies would be required to uncover potentially subtler differences in HMO concentrations and in the proportion of those with secretor status between these Asian ethnicities. Furthermore, we did not account for the potential influence of postpartum diet, such as traditional ethnically-related ‘confinement’ diets and dietary practices, which future studies should assess alongside HMO profiling. With a relatively modest sample of 71 (28.6%) participants providing data at both timepoints, caution is warranted in interpreting the temporal changes in HMO concentrations. Additionally, we did not assess breastmilk beyond 3-months since many women in our population returned to work at 4-months and 62% ceased breastfeeding by 6-months in our cohort. As the genotyping performed lacked depth, we were unable to assess the influence of more SNPs in the *FUT2* and *FUT3* genes on HMO profiles.

In summary, our study offers the first multi-ethnic Asian description of HMO profiles. It highlights the influence of genotype and maternity health conditions on HMO composition, and the consistency of secretor status and the stability of general HMO composition over the first 3 months postpartum across the three major Asian ethnicities of Chinese, Malay and Indian. Differences between findings in Asian and non-Asian studies cautions against extrapolating HMO understanding across populations. Our findings form a firm foundation upon which further research evaluating the implications of HMO composition for Asian infant health can be built. Ultimately, with an enhanced understanding of population-specific HMO biology, HMO-based applications could be developed to promote infant health and wellbeing.

## Data Availability

The data supporting the conclusions of this article will be made available upon reasonable request from the corresponding author. In addition, an appropriately qualified individual working in an appropriate institution where an institutional signatory can confirm the recipient’s adherence to relevant information safeguards stipulated in a formal Data Transfer Agreement.

## Footnotes

1
https://conda.io/

## References

[1] Bode L Raman AS Murch SH Rollins NC Gordon JI. Understanding the mother-breastmilk-infant “triad.”. Science. (2020) 367:1070–2. doi: 10.1126/science.aaw6147, 32139527

[2] Hill DR Chow JM Buck RH. Multifunctional benefits of prevalent HMOs: implications for infant health. Nutrients. (2021) 13:3364. doi: 10.3390/nu13103364, 34684364 PMC8539508

[3] Bode L Contractor N Barile D Pohl N Prudden AR Boons GJ . Overcoming the limited availability of human milk oligosaccharides: challenges and opportunities for research and application. Nutr Rev. (2016) 74:635–44. doi: 10.1093/nutrit/nuw025, 27634978 PMC6281035

[4] Chen X. Human milk oligosaccharides (HMOS): structure, function, and enzyme-catalyzed synthesis. Adv Carbohydr Chem Biochem. (2015) 72:113–90. doi: 10.1016/bs.accb.2015.08.002, 26613816 PMC9235823

[5] Soyyılmaz B Mikš MH Röhrig CH Matwiejuk M Meszaros-Matwiejuk A Vigsnæs LK. The mean of Milk: a review of human Milk oligosaccharide concentrations throughout lactation. Nutrients. (2021) 13:2737. doi: 10.3390/nu13082737, 34444897 PMC8398195

[6] McGuire MK Meehan CL McGuire MA. What’s normal? Oligosaccharide concentrations and profiles in milk produced by healthy women vary geographically. Am J Clin Nutr. (2017) 105:1086–100. doi: 10.3945/ajcn.116.139980 28356278 PMC5402033

[7] Jantscher-Krenn E von Schirnding L Trötzmüller M Köfeler H Kurtovic U Fluhr H . Human milk oligosaccharides are present in amniotic fluid and show specific patterns dependent on gestational age. Nutrients. (2022) 14:2065. doi: 10.3390/nu14102065, 35631205 PMC9146373

[8] Thum C Wall CR Weiss GA Wang W Szeto IMY Day L. Changes in HMO concentrations throughout lactation: influencing factors, health effects and opportunities. Nutrients. (2021) 13:2272. doi: 10.3390/nu13072272, 34209241 PMC8308359

[9] Plows JF Berger PK Jones RB Alderete TL Yonemitsu C Najera JA . Longitudinal changes in human Milk oligosaccharides (HMOs) over the course of 24 months of lactation. J Nutr. (2021) 151:876–82. doi: 10.1093/jn/nxaa427, 33693851 PMC8030713

[10] Azad MB Robertson B Atakora F Becker AB Subbarao P Moraes TJ . Human milk oligosaccharide concentrations are associated with multiple fixed and modifiable maternal characteristics, environmental factors, and feeding practices. J Nutr. (2018) 148:1733–42. doi: 10.1093/jn/nxy175, 30247646

[11] Soh SE Tint MT Gluckman PD Godfrey KM. Cohort profile: growing up in Singapore towards healthy outcomes (GUSTO) birth cohort study. Int J Epidemiol. (2014) 43:1401–9. doi: 10.1093/ije/dyt125, 23912809

[12] Ng S Aris IM Tint MT Gluckman PD Godfrey KM Shek LPC . High maternal circulating cotinine during pregnancy is associated with persistently shorter stature from birth to five years in an Asian cohort. Nicotine and Tobacco Research. (2019) 21:1103–12. doi: 10.1093/ntr/nty148, 30032178 PMC6211655

[13] Sampathkumar A Tan KM Chen L Chong MFF Yap F Godfrey KM . Genetic link determining the maternal-fetal circulation of vitamin D. Front Genet. (2021) 12:721488. doi: 10.3389/fgene.2021.721488, 34621292 PMC8490770

[14] McKinney W. Pandas: a foundational Python library for data analysis and statistics. Python for high performance and scientific computing. (2011) 14:1–9.

[15] Harris CR Millman KJ van der Walt SJ Gommers R. Array programming with NumPy. Nature. (2020) 585:357–62. doi: 10.1038/s41586-020-2649-2, 32939066 PMC7759461

[16] Seabold S Perktold J. Statsmodels: econometric and statistical modeling with python. SciPy. (2010) 7:92–6. doi: 10.25080/Majora-92bf1922-011

[17] Bode L Jantscher-Krenn E. Structure-function relationships of human milk oligosaccharides. Adv Nutr. (2012) 3:383S–91S. doi: 10.3945/an.111.001404, 22585916 PMC3649474

[18] Sprenger N Lee LY De Castro CA Steenhout P Thakkar SK. Longitudinal change of selected human milk oligosaccharides and association to infants’ growth, an observatory, single center, longitudinal cohort study. PLoS One. (2017) 12:e0171814. doi: 10.1371/journal.pone.0171814, 28182762 PMC5300226

[19] Sudarma V Sunardi D Marzuki NS Munasir Z Hidayat A Hegar B. Human milk oligosaccharide profiles and the secretor and Lewis gene status of Indonesian lactating mothers. Pediatric Gastroenterology, Hepatology & Nutrition. (2023) 26:266–76. doi: 10.5223/pghn.2023.26.5.266, 37736221 PMC10509021

[20] Ambalavanan A Chang L Choi J Zhang Y Stickley SA Fang ZY . Human milk oligosaccharides are associated with maternal genetics and respiratory health of human milk-fed children. Nat Commun. (2024) 15:7735. doi: 10.1038/s41467-024-51743-6, 39232002 PMC11375010

[21] Ren X Yan J Bi Y Shuttleworth PW Wang Y Jiang S . Human milk oligosaccharides are associated with lactation stage and Lewis phenotype in a Chinese population. Nutrients. (2023) 15:1408. doi: 10.3390/nu15061408, 36986137 PMC10059825

[22] Williams JE McGuire MK Meehan CL. Key genetic variants associated with variation of milk oligosaccharides from diverse human populations. Genomics. (2021) 113:1867–75. doi: 10.1016/j.ygeno.2021.04.004, 33831438

[23] Lefebvre G Shevlyakova M Charpagne A Marquis J Vogel M Kirsten T . Time of lactation and maternal fucosyltransferase genetic polymorphisms determine the variability in human milk oligosaccharides. Front Nutr. (2020) 7:574459. doi: 10.3389/fnut.2020.574459, 33195368 PMC7658960

[24] Wu J Wu S Huo J Ruan H Xu X Hao Z . Systematic characterization and longitudinal study reveal distinguishing features of human milk oligosaccharides in China. Current Developments in Nutrition. (2020) 4:nzaa113. doi: 10.1093/cdn/nzaa113 32734137 PMC7382630

[25] Zhou Y Sun H Li K Zheng C Ju M Lyu Y . Dynamic changes in human milk oligosaccharides in Chinese population: a systematic review and meta-analysis. Nutrients. (2021) 13:2912. doi: 10.3390/nu13092912, 34578788 PMC8464947

[26] Cheng YJ Yeung CY. Recent advance in infant nutrition: human milk oligosaccharides. Pediatrics & Neonatology. (2021) 62:347–53. doi: 10.1016/j.pedneo.2020.12.013, 33893051

[27] Newburg DS. Human milk oligosaccharides vary among populations. Am J Clin Nutr. (2017) 105:1027–8. doi: 10.3945/ajcn.117.155721, 28424182

[28] Fok D Aris IM Ho J Lim SB Chua MC Pang WW . A comparison of practices during the confinement period among Chinese, Malay, and Indian mothers in Singapore. Birth. (2016) 43:247–54. doi: 10.1111/birt.12233, 27018256 PMC4992357

[29] Austin S De Castro CA Sprenger N. Human milk oligosaccharides in the milk of mothers delivering term versus preterm infants. Nutrients. (2019) 11:1282. doi: 10.3390/nu11061282, 31195757 PMC6627155

[30] Thurl S Munzert M Boehm G Matthews C Stahl B. Systematic review of the concentrations of oligosaccharides in human milk. Nutr Rev. (2017) 75:920–33. doi: 10.1093/nutrit/nux044, 29053807 PMC5914348

[31] Wang X Liu J Li C Xu Y Wang X Lu Y . Pregnancy-related diseases and delivery mode can affect the content of human Milk oligosaccharides: a preliminary study. J Agric Food Chem. (2022) 70:5207–17. doi: 10.1021/acs.jafc.2c00147, 35434993

[32] Sawane K Takahashi I Ishikuro M Takumi H Orui M Noda A . Association between human Milk oligosaccharides and early adiposity rebound in children: a case–control study of the Tohoku medical megabank project birth and three-generation cohort study. J Nutr. (2025) 155:1498–507. doi: 10.1016/j.tjnut.2025.02.024, 40058699

[33] Pang WW Geddes DT Lai CT Michael N Huang J Chan YH . The prospective associations of fetal growth-related pregnancy complications with subsequent breastfeeding duration and markers of human milk production. Am J Clin Nutr. (2025) 121:478–87. doi: 10.1016/j.ajcnut.2024.11.008, 39542219

[34] Dou Y Luo Y Xing Y Liu H Chen B Zhu L . Human milk oligosaccharides variation in gestational diabetes mellitus mothers. Nutrients. (2023) 15:1441. doi: 10.3390/nu15061441, 36986171 PMC10059845

[35] Jincui H Dmitry G Carlito L Bruce GJ. Human milk secretory immunoglobulin a and lactoferrin N-glycans are altered in women with gestational diabetes mellitus. J Nutr. (2013) 143:1906–12. doi: 10.3945/jn.113.180695 24047700 PMC3827637

