# Supplementary material for: Human milk oligosaccharide composition, concentrations and association with maternal factors in the multi-ethnic Asian GUSTO cohort
Source: Front Nutr. 2026 May 28;13:1781871. doi: 10.3389/fnut.2026.1781871 (PMC13253375; doi:10.3389/fnut.2026.1781871)

**Supplementary Table 1.** Characteristics of participants providing breastmilk samples at 3-weeks and 3-months according to secretor status (defined by the relative abundance of 2’-FL and LNFP-I)

| Variables, n (%) | Week 3 | | Month 3 | |
| --- | --- | --- | --- | --- |
|  | Secretors  (*n* = 145) | Non-secretors  (*n* = 60) | Secretors  (*n* = 86) | Non-secretors  (*n* = 28) |
| Ethnicity  Chinese  Malay  Indian | 104 (71.7%)  24 (16.6%)  17 (11.7%) | 43 (71.7%)  9 (15.0%)  8 (13.3%) | 70 (81.4%)  8 (9.3%)  8 (9.3%) | 23 (82.1%)  3 (10.7%)  2 (7.1%) |
| Pre-pregnancy BMI (in kg/m^2^), Asian categories  Underweight (<18.5)  Normal (18.5 - < 23)  Overweight (23 - <27.5)  Obese (≥27.5) | 14 (10.3%)  75 (55.1%)  33 (24.3%)  14 (10.3%) | 7 (12.1%)  30 (51.7%)  14 (24.1%)  7 (12.1%) | 6 (7.8%)  44 (57.1%)  17 (22.1%)  10 (13.0%) | 3 (11.1%)  19 (70.4%)  5 (18.5%)  0 (0.0%) |
| Maternal level of physical activity before pregnancy  <600 MET-week  600-3000 MET-week  3000+ MET-week | 29 (20.1%)  80 (55.6%)  35 (24.3%) | 12 (20.3%)  31 (52.5%)  16 (27.1%) | 20 (24.4%)  41 (50.0%)  21 (25.6%) | 5 (17.9%)  18 (64.3%)  5 (17.9%) |
| Maternal level of physical activity during pregnancy  <600 MET-week  600-3000 MET-week  3000+ MET-week | 47 (32.6%)  72 (50.0%)  25 (17.4%) | 23 (39.0%)  26 (44.1%)  10 (16.9%) | 35 (42.2%)  33 (39.8%)  15 (18.1%) | 13 (46.4%)  9 (32.1%)  6 (21.4%) |
| Maternal smoking during pregnancy  Non-smoker  Plasma cotinine undetected but current smoker and/or ETS exposure  Plasma cotinine detectable, ≥0.17 µg/L | 86 (62.8%)  38 (27.7%)  13 (9.5%) | 35 (63.6%)  14 (25.5%)  6 (10.9%) | 57 (73.1%)  17 (21.8%)  4 (5.1%) | 21 (80.8%)  4 (15.4%)  1 (3.8%) |
| Pre-pregnancy alcohol consumption  No  Yes | 89 (61.4%)  56 (38.6%) | 31 (52.5%)  28 (47.5%) | 52 (61.2%)  33 (38.8%) | 15 (53.6%)  13 (46.4%) |
| Parity  Nulliparous  Parous | 70 (48.3%)  75 (51.7%) | 26 (43.3%)  34 (56.7%) | 37 (43.0%)  49 (57.0%) | 18 (64.3%)  10 (35.7%) |
| Gestational Diabetes (WHO-1999 definition^&^)  No  Yes | 119 (84.4%)  22 (15.6%) | 45 (80.4%)  11 (19.6%) | 68 (81.9%)  15 (18.1%) | 25 (92.6%)  2 (7.4%) |
| Fasting glucose at 26-28 weeks of pregnancy  Normal (<5.1 mmol/L)  High (≥5.1 mmol/L) | 135 (95.7%)  6 (4.3%) | 55 (98.2%)  1 (1.8%) | 80 (96.4%)  3 (3.6%) | 27 (100.0%)  0 (0.0%) |
| 2hr glucose (after 75g glucose load) at 26-28 weeks of pregnancy  Normal (<8.5 mmol/L)  High (≥8.5 mmol/L) | 128 (90.8%)  13 (9.2%) | 49 (87.5%)  7 (12.5%) | 75 (90.4%)  8 (9.6%) | 26 (96.3%)  1 (3.7%) |
| Hypertensive disorders of pregnancy#  No  Yes | 138 (95.2%)  7 (4.8%) | 53 (88.3%)  7 (11.7%) | 82 (96.5%)  3 (3.5%) | 27 (96.4%)  1 (3.6%) |
| Combined labor and mode of delivery  Non-labor cesarean section  Intrapartum cesarean section  Vaginal delivery | 14 (9.7%)  22 (15.2%)  109 (75.2%) | 5 (8.3%)  10 (16.7%)  45 (75.0%) | 8 (9.3%)  9 (10.5%)  69 (80.2%) | 4 (14.3%)  5 (17.9%)  19 (67.9%) |
| Gestational age at birth  Preterm (<37 weeks)  Early term (37^+0^ weeks to 38^+6^ weeks)  Term (39^+0^ weeks to 41^+6^ weeks) | 5 (3.4%)  63 (43.4%)  77 (53.1%) | 3 (5.0%)  20 (33.3%)  37 (61.7%) | 3 (3.5%)  41 (47.7%)  42 (48.8%) | 1 (3.6%)  4 (14.3%)  23 (82.1%) |
| Sex of child  Male  Female | 79 (54.5%)  66 (45.5%) | 23 (38.3%)  37 (61.7%) | 51 (59.3%)  35 (40.7%) | 10 (35.7%)  18 (64.3%) |
| Birthweight percentile (standardized for sex and gestation by local reference)  Small-for-Gestational-Age (SGA, <10^th^ centile)  Appropriately-Grown-for-Gestational-Age (AGA, 10-90^th^ centile)  Large-for-Gestational-Age (LGA, >90^th^ centile) | 12 (8.3%)  104 (71.7%)  29 (20.0%) | 8 (13.3%)  43 (71.7%)  9 (15.0%) | 5 (5.8%)  62 (72.1%)  19 (22.1%) | 2 (7.1%)  22 (78.6%)  4 (14.3%) |
| Full breastfeeding^1^ at 1 month  No  Yes | 85 (59.9%)  57 (40.1%) | 41 (69.5%)  18 (30.5%) | 39 (45.9%)  46 (54.1%) | 17 (60.7%)  11 (39.3) |
| Full breastfeeding at 3 months^  No  Yes | 92 (64.3%)  51 (35.7%) | 45 (76.3%)  14 (23.7%) | 43 (50.6%)  42 (49.4%) | 20 (71.4%)  8 (28.6%) |
| Any breastfeeding^2^ at 3 months  No  Yes | 26 (18.4%)  115 (81.6%) | 16 (28.6%)  40 (71.4%) | 0 (0.0%)  85 (100.0%) | 0 (0.0%)  27 (100.0%) |
| ^#^includes pre-eclampsia, eclampsia, superimposed pre-eclampsia on chronic hypertension, pregnancy-induced hypertension  ^Restricted to cases with milk samples collected at 3-months  ^1^Full breastfeeding includes those who were exclusively or predominantly breastfeeding, with no intake of solids; any method of breastmilk feeding is included (direct from the breast or expressed milk given through a bottle).  ^2^Any breastfeeding includes those who were breastfeeding, regardless of the extent of breastfeeding or the method of breastmilk feeding.  ^&^WHO-1999 definition of gestational diabetes with a 75g two-time point oral glucose tolerance test: fasting plasma glucose ≥7.0 mmol/L; 2-hour plasma glucose ≥7.8 mmol/L  Abbreviation: MET-week; Metabolic Equivalent Task per week  Note: Due to missing data, totals may not sum to the sample size shown at the top of each column. | | | | |

**Supplementary Table 2.** Absolute and relative HMO concentrations at 3-weeks and 3-months according to secretor status defined by the relative abundance of 2’-FL and LNFP-I

| **HMO** | **3-Weeks** | | | | **3-Months** | | | |
| --- | --- | --- | --- | --- | --- | --- | --- | --- |
|  | **Secretor** | | **Non-Secretor** | | **Secretor** | | **Non-Secretor** | |
|  | **Mean±SEM (nmol/mL)** | **Percentage (%)** | **Mean±SEM (nmol/mL)** | **Percentage (%)** | **Mean±SEM (nmol/mL)** | **Percentage (%)** | **Mean±SEM (nmol/mL)** | **Percentage (%)** |
| 2’-FL | 2391 ± 137 | 16.0 | 29 ± 5 | 0.3 | 1677 ± 114 | 10.4 | 28 ± 4 | 0.2 |
| 3-FL | 1410 ± 96 | 9.5 | 2270 ± 173 | 21.7 | 4878 ± 236 | 30.4 | 4967 ± 430 | 42.9 |
| LNnT | 221 ± 8 | 1.5 | 291 ± 24 | 2.8 | 220 ± 10 | 1.4 | 303 ± 66 | 2.6 |
| 3’SL | 437 ± 29 | 2.9 | 300 ± 16 | 2.9 | 680 ± 31 | 4.2 | 467 ± 42 | 4.0 |
| DFLac | 361 ± 14 | 2.4 | 24 ± 4 | 0.2 | 573 ± 29 | 3.6 | 31 ± 4 | 0.3 |
| 6’SL | 1396 ± 41 | 9.4 | 1122 ± 48 | 10.7 | 637 ± 31 | 4.0 | 514 ± 46 | 4.4 |
| LNT | 1782 ± 58 | 11.9 | 2395 ± 142 | 22.9 | 1277 ± 64 | 8.0 | 1553 ± 265 | 13.4 |
| LNFP-I | 2441 ± 99 | 16.4 | 151 ± 8 | 1.4 | 1423 ± 127 | 8.9 | 120 ± 15 | 1.0 |
| LNFP-II | 975 ± 63 | 6.5 | 2031 ± 121 | 19.4 | 1823 ± 60 | 11.4 | 2346 ± 170 | 20.3 |
| LNFP-III | 115 ± 5 | 0.8 | 94 ± 11 | 0.9 | 53 ± 4 | 0.3 | 57 ± 23 | 0.5 |
| LSTb | 135 ± 4 | 0.9 | 170 ± 11 | 1.6 | 134 ± 7 | 0.8 | 146 ± 14 | 1.3 |
| LSTc | 353 ± 14 | 2.4 | 211 ± 16 | 2.0 | 136 ± 8 | 0.8 | 57 ± 6 | 0.5 |
| DFLNT | 1522 ± 61 | 10.2 | 96 ± 19 | 0.9 | 1658 ± 77 | 10.3 | 113 ± 32 | 1.0 |
| LNH | 61 ± 3 | 0.4 | 67 ± 6 | 0.6 | 73 ± 5 | 0.5 | 45 ± 5 | 0.4 |
| DSLNT | 606 ± 21 | 4.1 | 580 ± 35 | 5.5 | 374 ± 21 | 2.3 | 326 ± 27 | 2.8 |
| FLNH | 241 ± 11 | 1.6 | 243 ± 22 | 2.3 | 136 ± 11 | 0.8 | 119 ± 15 | 1.0 |
| DFLNH | 200 ± 12 | 1.3 | 49 ± 5 | 0.5 | 72 ± 9 | 0.4 | 15 ± 2 | 0.1 |
| FDSLNH | 110 ± 7 | 0.7 | 248 ± 21 | 2.4 | 179 ± 13 | 1.1 | 309 ± 40 | 2.7 |
| DSLNH | 155 ± 8 | 1.0 | 105 ± 9 | 1.0 | 54 ± 4 | 0.3 | 51 ± 7 | 0.4 |

Abbreviations: SEM, Standard Error of the Mean; 2’-FL, 2’-fucosyllactose; 3-FL, 3-fucosyllactose; LNnT, lacto-N-neotetraose; 3’SL, 3’-sialyllactose; DFLac, difucosyllactose; 6’SL, 6’-sialyllactose; LNT, lacto-N-tetraose; LNFP, lacto-N-fucopentaose; LSTb, sialyllacto-N-tetraose b; LSTc, sialyllacto-N-tetraose c; DFLNT, difucosyllacto-N-tetraose; LNH, lacto-N-hexaose; DSLNT, disialyllacto-N-tetraose; FLNH, fucosyllacto-N-hexaose; DFLNH, difucosyllacto-N-hexaose; FDSLNH, fucosyldisialyllacto-N-hexaose; DSLNH, disialyllacto-N-hexaose

**Supplementary Figure 1. ﻿** Participant flowchart.


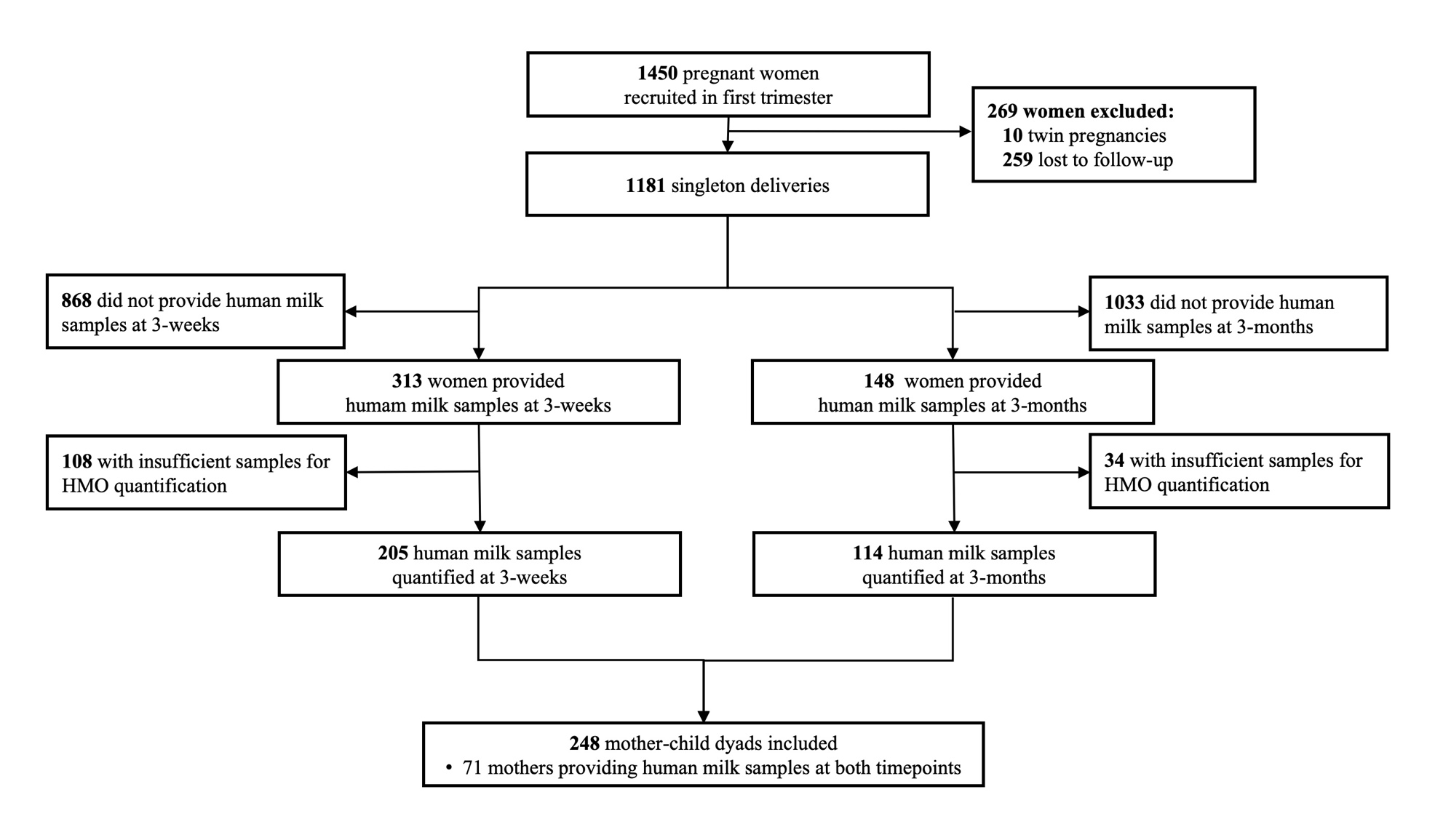

Supplement: Supplementary file 1 [file Table_1.DOCX]
